# Impact of metformin on melanoma: a meta-analysis and systematic review

**DOI:** 10.3389/fonc.2024.1399693

**Published:** 2024-05-23

**Authors:** Hua Feng, Shuxian Shang, Kun Chen, Xuan Sun, Xueping Yue

**Affiliations:** ^1^ Department of Dermatology, Beijing Tiantan Hospital, Capital Medical University, Beijing, China; ^2^ Hospital of Dermatology, Chinese Academy of Medical Sciences & Peking Union Medical College, Nanjing, China; ^3^ Interventional Neuroradiology Center, Beijing Tiantan Hospital, Capital Medical University, Beijing, China

**Keywords:** melanoma, metformin, survival outcome, safety outcomes, meta-analysis

## Abstract

**Background:**

There is evidence of a modest reduction in skin cancer risk among metformin users. However, no studies have further examined the effects of metformin on melanoma survival and safety outcomes. This study aimed to quantitatively summarize any influence of metformin on the overall survival (OS) and immune-related adverse effects (irAEs) in melanoma patients.

**Methods:**

Selection criteria: The inclusion criteria were designed based on the PICOS principles. Information sources: PubMed, EMBASE, Cochrane Library, and Web of Science were searched for relevant literature published from the inception of these databases until November 2023 using *‘Melanoma’* and *‘Metformin’* as keywords. Survival outcomes were OS, progression-free survival (PFS), recurrence-free survival (RFS), and mortality; the safety outcome was irAEs. Risk of bias and data Synthesis: The Cochrane tool for assessing the risk of bias in randomized trial 2 (RoB2) and methodological index for non-randomized studies (MINORS) were selected to assess the risk of bias. The Cochrane Q and *I* ^2^ statistics based on Stata 15.1 SE were used to test the heterogeneity among all studies. Funnel plot, Egger regression, and Begg tests were used to evaluate publication bias. The leave-one-out method was selected as the sensitivity analysis tool.

**Results:**

A total of 12 studies were included, involving 111,036 melanoma patients. The pooled HR for OS was 0.64 (95% CI [0.42, 1.00], p = 0.004, I^2 =^ 73.7%), HR for PFS was 0.89 (95% CI [0.70, 1.12], p = 0.163, I^2 =^ 41.4%), HR for RFS was 0.62 (95% CI [0.26, 1.48], p = 0.085, I^2 =^ 66.3%), and HR for mortality was 0.53 (95% CI [0.46, 0.63], p = 0.775, I^2 =^ 0.0%). There was no significant difference in irAEs incidence (OR = 1.01; 95% CI [0.42, 2.41]; p = 0.642) between metformin and no metformin groups.

**Discussion:**

The improvement in overall survival of melanoma patients with metformin may indirectly result from its diverse biological targets and beneficial effects on multiple systemic diseases. While we could not demonstrate a specific improvement in the survival of melanoma patients, the combined benefits and safety of metformin for patients taking the drug are worthy of recognition.

**Systematic review registration:**

https://www.crd.york.ac.uk/PROSPERO/, identifier CRD42024518182.

## Introduction

Metformin was introduced in Europe and America between the 1950s and 1990s as a treatment for diabetes (1). At present, metformin is one of the most popular oral hypoglycemic drugs, and as a first-line treatment drug, it is considered the ideal initial treatment for type 2 diabetes mellitus (T2DM) patients (2, 3). In recent years, numerous studies have identified metformin therapy as a possible protective factor for cancer in patients with T2DM (4), including skin cancer (5), pancreatic cancer (6), colorectal cancer (7), bladder cancer (8), etc. At the same time, as T2DM has been identified as a risk factor for many cancers (colorectal, breast, endometrial, and pancreatic cancers, among others) (4), there is still much interest in the cancer prevention effects of anti-diabetes treatments, although the results of different studies are disputed (9). The mechanism of anti-cancer action of metformin is still inconclusive, and pharmacoepidemiological studies are still the main source of evidence. Possible hypotheses are that metformin inhibits the growth of certain tumors through cellular autonomic mechanisms, secondary inhibition of AMP kinase activation and protein synthesis, decreased insulin and insulin growth factor-1 signaling, and inhibition of reactive oxygen species (ROS) production and somatic mutations (10, 11).

The incidence of cutaneous melanoma has been growing, and the United States Surveillance, Epidemiology, and End Results Program (SEER) reported 97,610 new cases in 2023, making it the fourth most common type of cancer and accounting for 5.0% of new cancer cases (12). Research on skin melanoma has also been making significant progress. While previous studies have only identified obesity and metabolic syndrome as potential risk factors for melanoma (13), a cross-sectional multicenter study involving 443 patients published in 2021 demonstrated that T2DM is associated with more aggressive cutaneous melanoma (14). Data based on tumor mouse models published the following year further confirmed that T2DM may be associated with melanoma aggressiveness (15), making the cancer prevention effects of diabetes drugs an even more valuable topic. Only one meta-analysis based on 4 randomized controlled trials and observational studies published in 2020 reported no statistically significant associations between metformin and the risk of melanoma (9). Since then, multiple prospective studies conducted in Sweden, Russia, the United Kingdom (UK), Germany, and the United States (US) have again examined the effects of metformin on melanoma survival and safety outcomes (5, 16–19). Some of these revealed a positive effect of metformin use on the prognosis of melanoma (5, 18, 19). Moreover, valuable arguments such as the treatment regimen of metformin combined with dacarbazine (16) and the effect of metformin on the efficacy of pembrolizumab in resected stage III melanoma (17) have also been validated by practice.

Therefore, we believe that, as an update and supplement to the previous meta-analysis, it is necessary to research relevant literature, increase the aggregation of newly published research results, perform meta-analyses, and comprehensively synthesize evidence to further summarize any influence of metformin on the survival and safety outcomes of melanoma.

## Methods

### Literature search

This meta-analysis was performed based on the Preferred Reporting Items for Systematic Reviews and Meta-Analyses guidelines (PRISMA 2020) (20). (PROSPERO registration number CRD42024518182, https://www.crd.york.ac.uk/PROSPERO/display_record.php?RecordID=518182) Literature search followed the PICOS principle. PubMed, Embase, Web of Science and Cochrane Library databases were systematically searched for all eligible studies published from database inception until November 2023. MeSH terms “Melanoma”, “Metformin” were used as keywords; other relevant keywords were also searched. Specific search strategies are shown in **Supplementary Table S1**. Two independent researchers performed literature search and selection. Any discrepancies in the search process were resolved through discussion.

### Eligibility criteria

The inclusion criteria were as follows: (1) research targeting patients clinically diagnosed with melanoma; (2) the intervention group or control group received metformin monotherapy or combination therapy; (3) study endpoints included any one of melanoma OS, PFS, RFS, mortality, irAEs, incidence, and ORR; (4) single-arm studies, randomized controlled trials (RCTs), and observational studies using metformin as an intervention were included; (5) full text of the study was available. The exclusion criteria were: (1) conference papers, reviews, case reports, editorials, dissertations, and chapters in handbooks were excluded; (2)1) duplicate published studies.

### Data extraction

Two independent authors extracted information. Differences arising from the data extraction process were resolved by discussion. Data included the authors of the articles, publication year, study design, study location, sample size, mean age, female proportion, number of participants with different cancer stages (I/II/III-IV), intervention and control, follow-up period, and study outcomes. Survival outcomes were OS, PFS, RFS, and mortality; safety outcomes were irAEs, and exploratory outcomes were incidence and ORR.

### Quality assessment

We selected two different scales for quality assessment according to the different study designs of the included studies. The RoB2 (21) was selected to assess the risk of bias and quality of evidence of the 2 included RCTs. The MINORS (22) was used to evaluate the potential bias and quality of 10 non-randomized trials. Two different investigators carried out the quality assessment.

RoB2 was used to evaluate the bias involved in the following processes: randomization, deviations from expected interventions, missing outcome data, outcome measurements, and outcome selection. MINORS consist of a total of 12 programs, requiring a study with a clear purpose, inclusion of consecutive patients, prospective data collection, selection of endpoints appropriate for the purpose of the study, unbiased evaluation of the endpoints, matching the primary endpoint for the follow-up period, follow-up loss < 5%, and prospective calculation of the sample size. Articles 9 through 12 are additional criteria for evaluating control studies, including selection of the gold standard as intervention for the control group, baseline equivalence between groups, and statistical analysis consistent with the study design. In the case of a total of 24 points, each entry is 0–2 points. 0 indicates that the relevant information is not provided. A score of 1 indicates insufficient information. A score of 2 indicates adequate information.

### Statistical analysis

All analyses were performed using the Stata 15.1 SE version. Hazard ratios (HRs), odds ratios (ORs), and their corresponding 95% confidence intervals (CIs) were used to compare the outcomes. Studies providing the number of melanoma cases and incidence were separated for pooled analysis. Cochran’ s Q-test and the *I^2^* index were selected to calculate statistical heterogeneity among included studies (Q-test P>0.10 and *I^2^* >50% represented high heterogeneity). Random effects models were used to analyze variables with high inter-study heterogeneity. The fixed effects model was used for variables with low heterogeneity. A P-value < 0.05 was used as the threshold for statistical significance. In order to evaluate potential confounding effects and the robustness of the combined results, the leave-one method was chosen as the sensitivity analysis tool. If the pooled results after the exclusion of a study were inconsistent with the original pooled ones, the study was excluded as a potential confounder. Subgroup analysis was conducted to examine the OS, PFS and ORR in different study designs (RCT, prospective, or retrospective design), age (mean age ≤ 60, or >60), and proportion of participants with stage iii-iv tumors (≤ 50, or >50). Potential publication bias was identified by funnel plot, Egger regression test, and Begg test.

## Results

### Study selection and study characteristics

**Figure 1:**
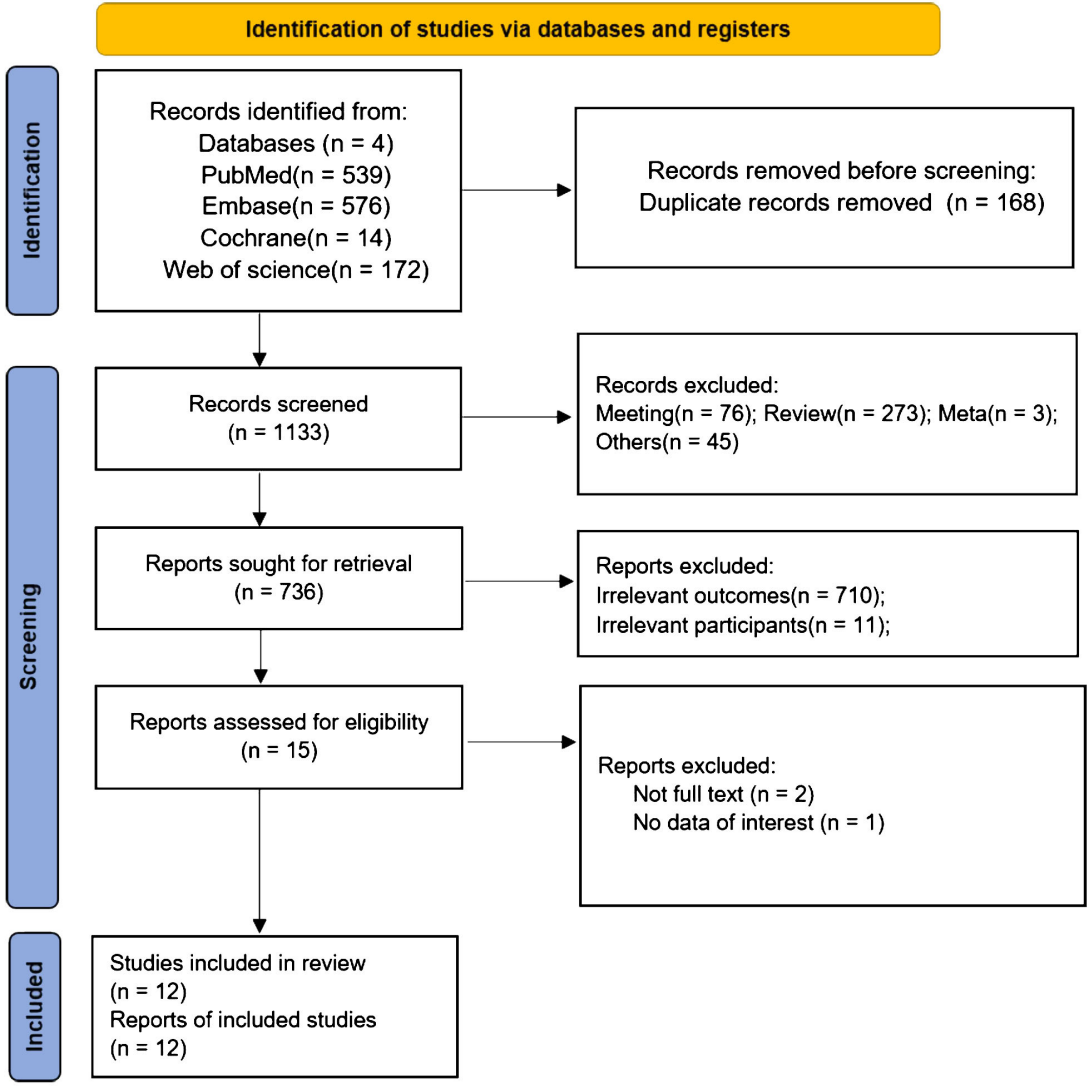
Flowchart of the study selection.

The study selection process is shown in **Figure 1**. The initial search resulted in 1301 records, with 168 records marked as duplicates. A total of 1118 records not related to the topic of this research article were excluded after reviewing the title and abstract, and 3 records were excluded after reviewing the full text of 15 articles. Among these, the full text was unavailable for 2 studies, and 1 study did not have the right outcomes. Therefore, 12 studies were included in this meta-analysis (5, 16–19, 23–29).

The characteristics of the included studies are presented in **Table 1**. There were 111,036 melanoma patients included in this meta-analysis, 74,060 of whom were treated with metformin. Studies were conducted in 9 countries, including America (n=3), England (n = 2), China (n=1), Sweden (n=1), Russia (n=1), Greece (n=1), Lithuania (n=1), Lebanon (n=1), and Germany (n = 1). There were 9 included studies with retrospective study design, 2 with RCT design, and 1 with prospective design. The mean age of the included melanoma patients ranged from 57.6 to 73.4 years. Female sex proportion ranged from 31.8% to 65.18%. The number of patients with stage I, stage II, and stage III-IV melanoma included in the analysis was 14,072, 7139, and 15, 253, respectively. The types of control group interventions included no metformin (n = 10), rosiglitazone (n = 1), and melatonin (n = 1), although they were not included in the analysis. Patients in the original study were followed up for 4.1 - 42.4 years. Three of the original studies included participants with type 2 diabetes (T2D), five studies with melanoma, two studies with melanoma combined with T2D, and two studies did not restrict whether melanoma patients had T2D. As the definitions of metformin use differed widely in the original studies, we summarized the original text in **Table 2**.

**Table 1:** Characteristics of the included studies.

Author, Year	country	Study design	Sample size			Mean age	Female (%)	Number of cancer stages			Intervention	Control	Follow-up duration	Outcomes
			Total	Intervention	Control			I	II	III-IV				
Home 2010 (23)	England	RCT	2225	1122	1103	/	/	/	/	/	Metformin	Rosiglitazone	/	Number of people and HR: Developing melanoma
Krakowski 2023 (5)	Sweden	Prospective study	1162	588	574	73.4	36.8	766	316	80	Metformin	no Metformin	4.1 years	HR: OS, Death, MSS
Wang 2020 (24)	USA	Retrospective study	330	330		60	37	42	50	234	Metformin	no Metformin	/	HR: OS, PFS
Novik 2021 (16)	Russia	RCT	57	38	19	/	/	0	3	54	Metformin	Melatonin	/	number of people: ORR
Kennedy 2023 (17)	England	Retrospective study	1019	965	54	/	38.4	0	0	1019	Metformin	no Metformin	42.4 months	HR: RFS, DMFS
Tseng 2017 (25)	China	Retrospective study	32474	16237	16237	59.28	42.49	12692	6408	13374	Metformin	no Metformin	/	Number of people and HR: Developing melanoma
Tsilidis 2014 (26)	Greece	Retrospective study	69748	51484	18264	/	56.59	/	/	/	Metformin	Sulfonylurea	5.1 years	HR: Developing melanoma
Urbonas 2020 (27)	Lithuania	Retrospective study	2757	2654	103	58.74	65.18	55	69	28	Metformin	no Metformin	/	HR: Death, MSM
Afzal 2018 (28)	Lebanon	Retrospective study	55	22	33	63.44	38.18	33	22	10	Metformin	no Metformin	/	HR: OS, PFS; number of people: ORR,irAEs
Spoerl 2022 (18)	Germany	Retrospective study	382	39	343	/	41.88	274	88	20	Metformin	no Metformin	4.5 years	HR: RFS
Failing 2016 (29)	USA	Retrospective study	159	159		57.6	42	0	0	159	Metformin	no Metformin	/	HR: OS, PFS; number of people: ORR, irAEs
Augustin 2023 (19)	USA	Retrospective study	668	422	246	64.2	31.8	210	183	275	Metformin	no Metformin	/	HR: OS, PFS

**Table 2:** Definition of metformin use.

study	Diagnosis	Definition of metformin use
Home 2010 (23)	T2D	/
Krakowski 2023 (5)	T2D+melanoma	metformin use as patients with at least one dispensation 6 months prior to or after CM diagnosis.
Wang 2020 (24)	melanoma	metformin use at baseline and within 6 weeks of anti-PD-1 treatment initiation
Novik 2021 (16)	melanoma	dacarbazine on day 1 of a 28-day cycle with melatonin
Kennedy 2023 (17)	melanoma	
Tseng 2017 (25)	T2D	/
Tsilidis 2014 (26)	T2D	metformin within 12 months of their diagnosis
Urbonas 2020 (27)	melanoma	oral administration of any formulation comprising metformin or a salt thereof between the date of randomisation and 30 days thereafter
Afzal 2018 (28)	melanoma ± T2D	ipilimumab, nivolumab, and/or pembrolizumab plus metformin
Spoerl 2022 (18)	melanoma ± T2D	/
Failing 2016 (29)	melanoma	/
Augustin 2023 (19)	T2D+melanoma	/

T2D, Type 2 Diabetes Mellitus.

PD-1, Programmed death 1.

### Study quality

The risk of bias assessment results of the included studies are shown in **Tables 2**, **3**. Two RCT studies using RoB2 for quality assessment were both evaluated as having a low risk of bias (**Table 3**). Of the 10 non-randomized studies using MINORS to evaluate quality, 8 included controls, and 2 were without controls. All 10 studies received high-quality evaluation scores, 23 for controlled and 15 for no-controlled studies. The only consideration for the 10 non-randomized studies was the relatively short follow-up duration, which was inappropriate for the primary endpoint (**Table 4**).

**Table 3:** Quality assessment results of the included RCTs.

Study	D1	D2	D3	D4	D5	Overall
Home 2010 (23)	Low	Low	Low	Low	Low	Low
Novik 2021 (16)	Low	Low	Low	Low	Low	Low

D1: Bias arising from the randomization process;

D2: Bias due to deviations from intended intervention;

D3: Bias due to missing outcome data;

D4: Bias in measurement of the outcome;

D5: Bias in the selection of the reported results.

**Table 4:** Quality assessment results of the included non-RCTs.

Study	T1	T2	T3	T4	T5	T6	T7	T8	T9	T10	T11	T12	Total
Krakowski 2023 (5)	2	2	2	2	2	1	2	2	2	2	2	2	23
Wang 2020 (24)	2	2	2	2	2	1	2	2					15
Kennedy 2023 (17)	2	2	2	2	2	1	2	2	2	2	2	2	23
Tseng 2017 (25)	2	2	2	2	2	1	2	2	2	2	2	2	23
Tsilidis 2014 (26)	2	2	2	2	2	1	2	2	2	2	2	2	23
Urbonas 2020 (27)	2	2	2	2	2	1	2	2	2	2	2	2	23
Afzal 2018 (28)	2	2	2	2	2	1	2	2	2	2	2	2	23
Spoerl 2022 (18)	2	2	2	2	2	1	2	2	2	2	2	2	23
Failing 2016 (29)	2	2	2	2	2	1	2	2					15
Augustin 2023 (19)	2	2	2	2	2	1	2	2	2	2	2	2	23

T1: A stated aim of the study.

T2: Inclusion of consecutive patients.

T3: Prospective collection of data.

T4: Endpoint appropriate to the study aim.

T5: Unbiased evaluation of endpoints.

T6: Follow-up period appropriate to the major endpoint.

T7: Loss to follow-up not exceeding 5%.

T8: Prospective calculation of the sample size.

T9: A control group having the gold standard intervention.

T10: Contemporary groups.

T11: Baseline equivalence of groups.

T12: Statistical analyses adapted to the study design.

### Overall survival

**Figure 2:**
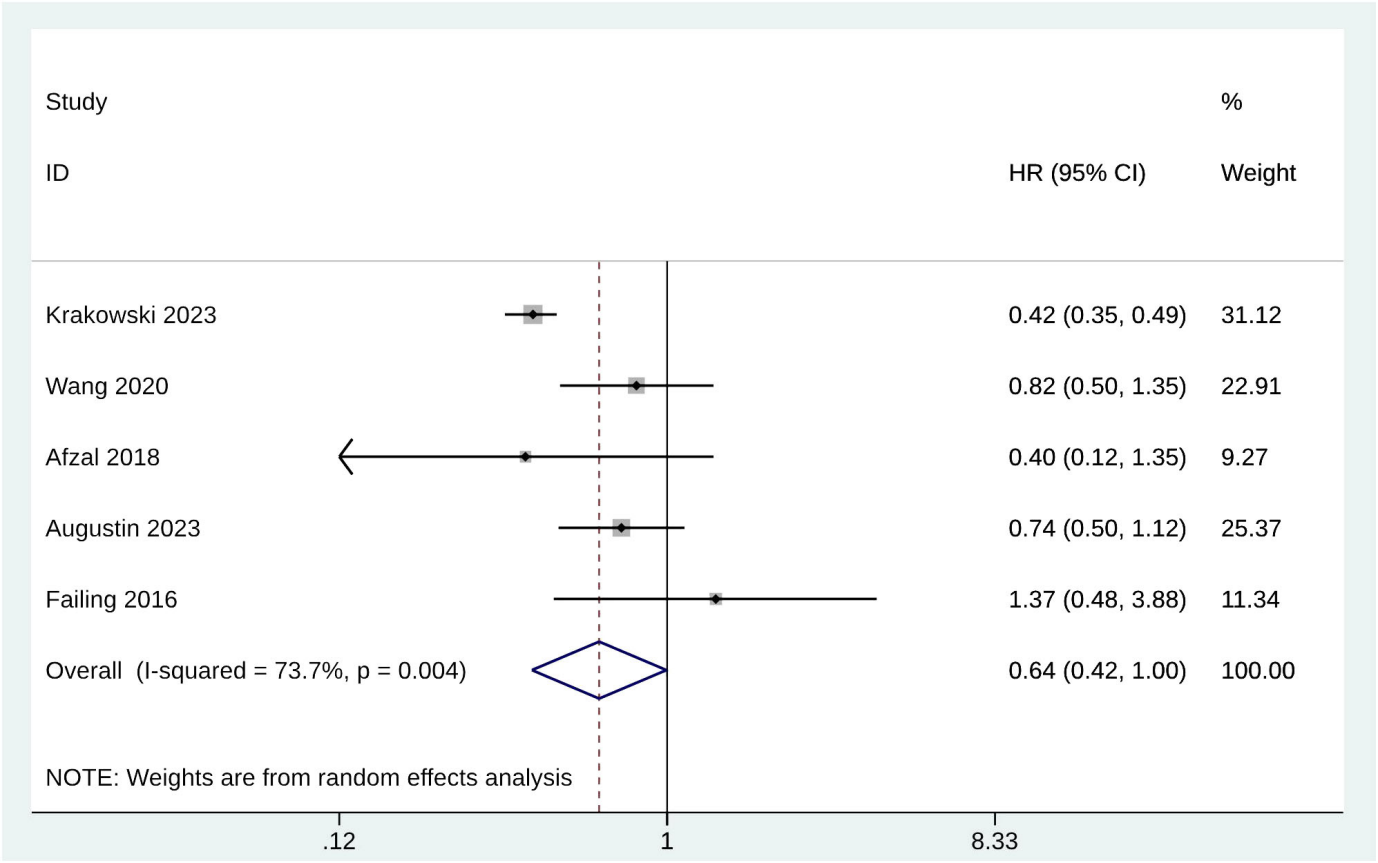
Forest plot of OS.

HR, lower confidence interval (LCI), and upper confidence interval (UCI) of melanoma patients’ OS were extracted from the original text to summarize the effect of metformin therapy on overall survival endpoints. The original OS data were obtained from 1521 melanoma patients in 5 studies. The analysis results showed that for patients receiving metformin treatment, the pooled HR for OS was 0.64 with significant heterogeneity (95% CI [0.42, 1.00], p = 0.004, I^2^= 73.7%) (**Figure 2**). The sensitivity analysis showed that the meta-analysis results were not robust, and the pooled results increased significantly when Krakowski 2023 was excluded (**Supplementary Figure S1**).

### Progression-free survival

**Figure 3:**
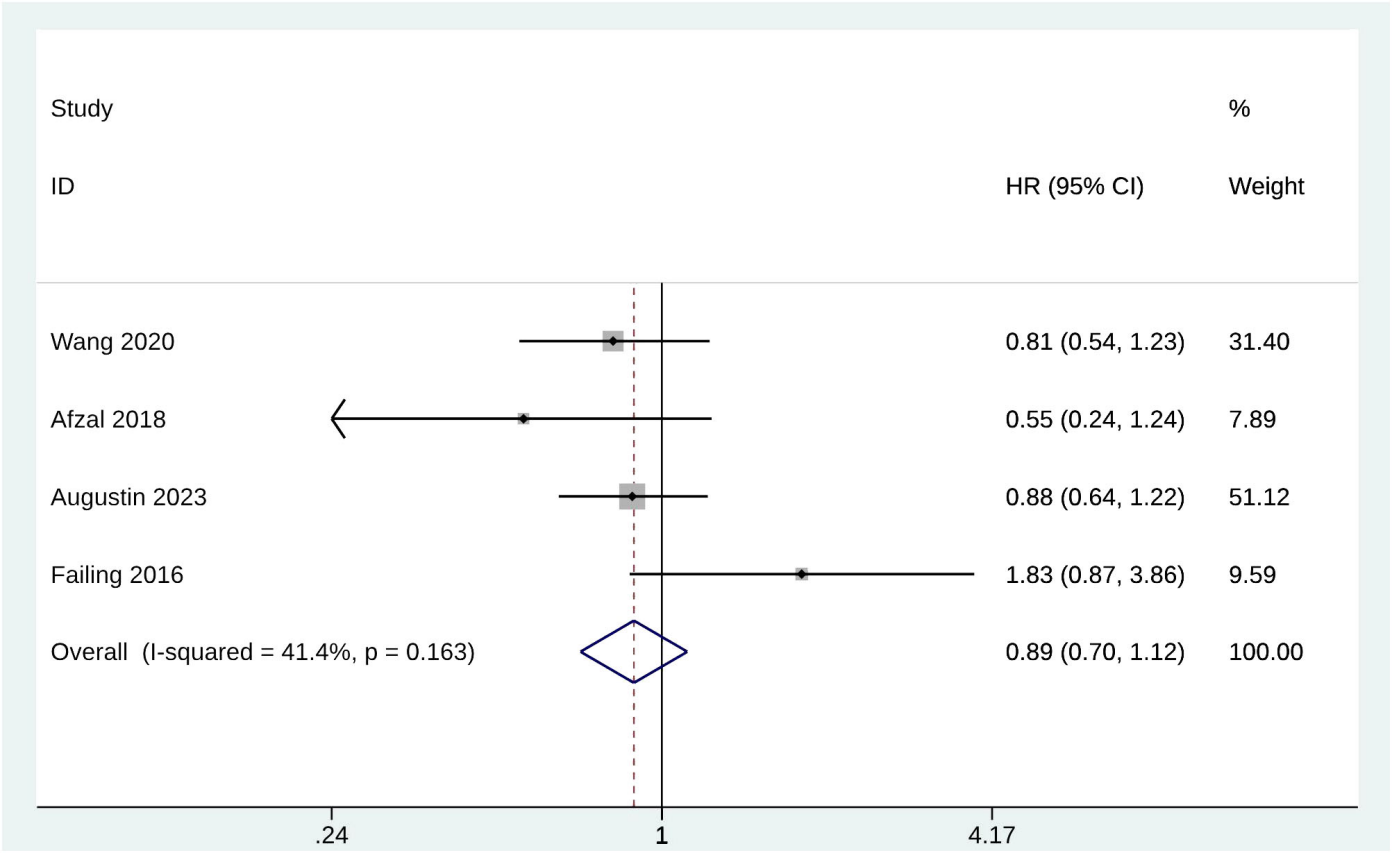
Forest plot of PFS.

There were 4 studies with 933 melanoma patients who reported PFS after metformin treatment. HR was used to compare PFS between groups. The pooled HR for PFS for patients receiving metformin treatment was 0.89 (95% CI [0.70, 1.12], p = 0.163, I^2^ = 41.4%) (**Figure 3**). Heterogeneity was not significant; the fixed-effects model was used for the analysis. Sensitivity analysis demonstrated that Failing 2016 may be the potential confounder since a significant reduction of the pooled PFS was observed after excluding this study (**Supplementary Figure S2**).

### Recurrence-free survival

Two studies counted RFS in 1004 melanoma patients treated with metformin. The pooled HR for RFS was 0.62 with significant heterogeneity (95% CI [0.26, 1.48], p = 0.085, I^2^= 66.3%) (**Supplementary Figure S3**). Because there were only two studies with considerable heterogeneity, the sensitivity analysis results were of no reference value (**Supplementary Figure S4**).

### Mortality

**Figure 4:**
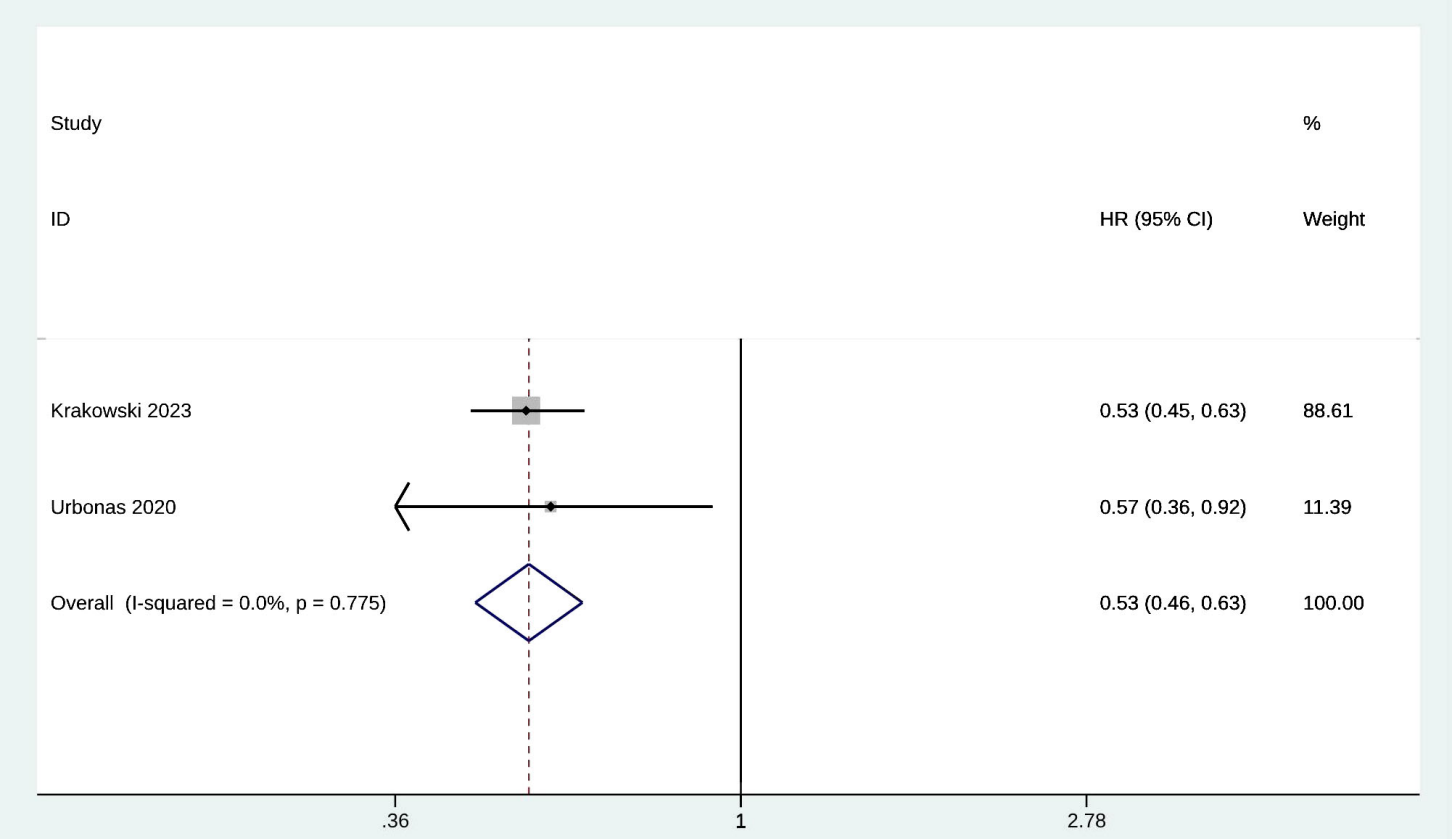
Forest plot of mortality.

Mortality was evaluated by 2 of the included studies with 3242 patients treated with metformin. The pooled HR for mortality was 0.53 (95% CI [0.46, 0.63], p = 0.775, I^2^= 0.0%) (**Figure 4**). Heterogeneity was not significant; the fixed-effects model was used for analysis. The sensitivity analysis results were robust (**Supplementary Figure S5**).

### Complications

**Figure 5:**
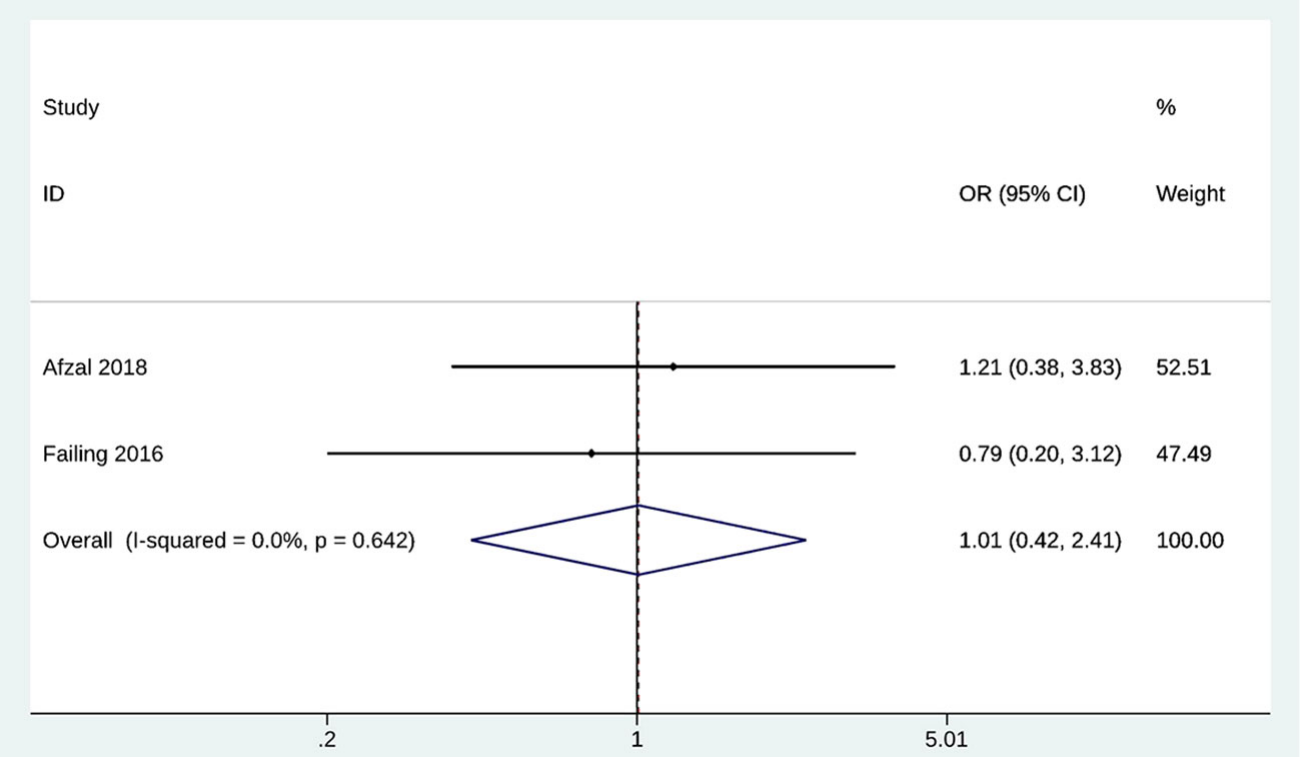
Forest plot of irAEs.

In this part, we mainly focused on the difference in the number of immune-related adverse effects after metformin treatment. IrAEs were evaluated by 2 of the included studies with 181 patients treated with metformin. No significant risk difference in irAEs incidence was observed between metformin and no metformin groups (OR = 1.01; 95% CI [0.42, 2.41]; p = 0.642) (**Figure 5**). Heterogeneity was not significant; the fixed-effects model was used for analysis (I2 = 0.00%). Therefore, no significant heterogeneity was observed in the sensitivity analysis (**Supplementary Figure S6**).

### Incidence

We evaluated the incidence of melanoma in patients treated with metformin by comparing the number of cases and the HR value of incidence, respectively. There were 2 studies that compared the number of melanoma cases between metformin and no metformin groups. Other 3 trials with 68843 patients reported the HR of melanoma incidence. No significant difference in the number of melanoma cases was observed (OR = 0.84; 95% CI [0.40, 1.78]; p = 0.007, I^2^ = 86.3%) (**Supplementary Figure S7**), with the HR of incidence was 0.94 (95% CI [0.55, 1.61]; p = 0.005, I^2^= 81.3%) (**Supplementary Figure S8**). Heterogeneity was significant, and the random-effects model was used for analysis. Tseng 2017 may be a potential source of heterogeneity in the pooled analysis of HR for incidence (**Supplementary Figures S9**, **10**).

### Objective response rate

ORR was evaluated by 3 of the included studies with 219 patients treated with metformin. There was no significant difference in ORR in the metformin group compared with the melatonin or no metformin group (OR = 0.92; 95% CI [0.41, 2.08], p = 0.208) (**Supplementary Figure S11**). Heterogeneity was not significant, so the fixed-effects model was used for analysis (I2 = 36.3%). Sensitivity analysis suggested that Afzal 2018 may be a potential source of heterogeneity (**Supplementary Figure S12**).

### Study design subgroup-analysis

In view of the heterogeneity of the original data sources, subgroup analysis divided the included studies into RCT design, prospective design, and retrospective design groups. Considering the number of studies, subgroup analyses were performed for OS and ORR.

In prospective design subgroups, the HR for OS rate in the metformin group was 0.42 (95% CI [0.35, 0.50], n = 1), and 0.78 in retrospective design subgroups (95% CI [0.58, 1.04], p = 0.498, I^2^= 0.00%, n = 4). It was also reconfirmed that Krakowski 2023 may be the source of heterogeneity for OS analysis (**Supplementary Figure S13**).

In both RCT design and retrospective design subgroups, ORR was not significantly different between groups (OR = 0.23; 95% CI [0.02, 2.71], n = 1; OR = 1.11; 95% CI [0.46, 2.64], p = 0.193, I^2^= 40.9%, n = 6) (**Supplementary Figure S14**).

### Age subgroup-analysis

Stratified by the average age of enrolled patients, the study was divided into ≤60 and >60 subgroups, subgroup analyses were performed for OS and PFS.

The pooled HR of OR analysis in ≤60 subgroup was different from the original meta-analysis results (≤60: HR = 0.90; 95% CI [0.58, 1.41], p = 0.650; >60: HR = 0.52; 95% CI [0.33, 0.82], p = 0.005). The pooled HR analysis result remain unchanged in PFS (>60: HR = 0.83, 95% CI [0.61, 1.12], p = 0.213, I^2^= 71.6%; ≤60: HR = 0.98, 95% CI [0.68, 1.41], p = 0.913, I^2^ = 8.3%).

Significant heterogeneity reduction was observed in OR and mean age >60 subgroups of PFS (**Supplementary Figures S15**, **16**). We suggest that age is a potential source of heterogeneity.

### Proportion of stage iii-iv subgroup-analysis

Considering that tumor stage is an important factor affecting the therapeutic effect, we divided the original study into two subgroups, > 50% and portent factor affecting the therapeutic effect, we divided the original study in the clinical trials. The subgroup analyses were performed for OS, PFS and ORR.

The pooled HR of OR analysis in proportion of stage iii-iv > 50% subgroup was different from the original meta-analysis results (> 50%: HR = 0.90; 95% CI [0.58, 1.41], p = 0.650; ≤50%: HR = 0.52; 95% CI [0.33, 0.82], p = 0.005). The pooled analysis result remain unchanged in PFS (>50%: HR = 0.98, 95% CI [0.68, 1.41], p = 0.913, I^2^= 71.6%; ≤50%: HR = 0.83, 95% CI [0.61, 1.21], p = 0.213, I^2^= 8.3%) and ORR (>50%: OR = 0.4, 95% CI [0.1, 1.56], p = 0.187, I^2^= 0.0%; ≤50%: OR = 1.79, 95% CI [0.58, 5.52], p = 0.314, n = 1).

Significant heterogeneity reduction was observed in OR, proportion of stage iii-iv ≤ 50% subgroups of PFS, and ORR (**Supplementary Figures S15**, **16**). We suggest that tumor stage is a potential source of heterogeneity. However, significant heterogeneity increase was found in proportion of stage iii-iv > 50% subgroups of PFS (**Supplementary Figure S17**-**19**).

### Publication bias and sensitivity analysis

Publication bias was assessed using the funnel plot, Egger regression test, Begg test, and trim and fill method. According to the funnel plot, Egger regression test, and Begg test results, potential publication bias was observed in OS and PFS analysis (**Supplementary Figures S20**-**27**). However, the trim and fill method did not identify any studies that might be missing.

The results of the sensitivity analysis showed that Krakowski 2023 may be the source of heterogeneity in OS, Failing 2016 was identified as the potential confounder of PFS, Tseng 2017 as a potential source of heterogeneity in the pooled analysis of HR for incidence, and Afzal 2018 as a potential confounder for ORR.

## Discussion

Metformin, whose hypoglycemic mechanism works by inhibiting the production of glucose in the liver, is a first-line treatment for type 2 diabetes and one of the most commonly prescribed drugs globally, taken by nearly 200 million patients worldwide (30). As an “old drug” that has been widely used in clinical practice, the mechanism of its therapeutic effect has always been controversial, and the related research results of new mechanisms have emerged in an endless stream. The latest mechanistic findings suggest that the hypoglycemic effects of metformin are not only due to its exclusive role in the liver but that sites of extrahepatic action, particularly on the gut and its microbiota, are involved in the play of its various clinical benefits (31). In addition, metformin has been shown to have immunomodulatory properties in a variety of pathological settings, directly or indirectly involved in the regulation of host innate and adaptive immune responses, including cancer (32), high-inflammatory diseases (33), and certain infectious diseases (e.g., tuberculosis and COVID-19) (34).

Based on the progress of research on new targets and mechanisms of action of metformin, the target diseases of pharmacoepidemiological studies on this drug have gradually increased. The efficacy of metformin in various diseases has been confirmed by clinical studies, making metformin regarded as a “miracle drug”; its benefits include anti-aging, treatment of cognitive impairment, anti-cancer, and cardiovascular disease improvement (31). The most important benefit is its preventive effect in people at high risk for T2DM, with more than 60% of the effect attributed to its ability to sustain weight loss due to increased circulating levels of GDF15 (35). Meanwhile, metformin is currently considered an anti-aging drug because it has been shown to extend the median and maximum life span in studies conducted in several species, including C. elegans (36), drosophila (37), rodents (38), and humans (39). It is believed that metformin achieves its anti-aging properties through the evolutionary conservation of microbial-derived metabolites (40), changing the human intestinal microbiota (41), and delaying the occurrence of immune aging and related inflammation (42).

The first known study that observed metformin reducing cancer risk was published in 2005 (43). Subsequently, the mechanism of action of metformin therapy in preventing the onset and prognosis of various cancers has also been extensively studied. *In vitro* and preclinical studies suggest that metformin has antitumor effects and inhibits tumor growth by inhibiting mitochondrial OXPHOS, which are associated with AMPK-dependent and AMPK-independent mechanisms (44). However, it is important to note that not all studies supported the protective effect of metformin on cancer risk. A small open-label study involving 17 patients with advanced melanoma showed no benefit of 1 g metformin given 3 times daily after first-line treatment progression (45). In a retrospective study that included 55 patients with advanced melanoma treated with checkpoint inhibitors, patients treated with metformin had longer overall survival; however, the results were not significant due to the small sample size (HR 0.40, 95% CI 0.12–1.35) (28). Another large sample study included 1,162 patients with T2MD and stage I-IV melanoma; although metformin was shown to extend OS, no changes in melanoma-specific survival were observed (5). This is consistent with our analysis, where melanoma patients treated with metformin all achieved longer survival and lower mortality compared with the non-metformin group. However, only the efficacy advantage on OS was statistically significant, and younger age and higher tumor stage have a negative impact on the efficacy of metformin in extending OS. There was no difference in immune safety outcomes between metformin and other treatments. Metformin did not have a significant advantage in improving melanoma incidence or ORR. High heterogeneity was observed in the analyses of OS, RFS, and incidence, with different study designs, follow-up duration, and intervention modalities in the control group potentially representing the sources of heterogeneity.

These results also lead us to consider that metformin may indirectly affect disease survival through its protective effects on cell metabolism, anti-hyperglycemia, enhanced insulin sensitivity, reduced oxidative stress, and cardiovascular function. The results of a literature review published in 2021 examining the effects of metformin on aging, healthy lifespan, and longevity in humans and other species showed that metformin can reduce early mortality associated with various diseases and, therefore, can improve healthy lifespan, extending the length of overall healthy life (46). The results of two previous large controlled studies also confirmed that diabetes patients using metformin had a similar risk of cancer compared with diabetes patients using sulfonylureas (Home 2010, Tsilidis 2014). The combination study also showed that metformin did not increase the efficacy of systemic chemotherapy for melanoma (Novik 2021). Therefore, until pharmacoepidemiological studies can further prove the specific improvement efficiency of metformin on tumor survival or morbidity, or pharmacokinetic studies make breakthroughs in the mechanism of action of metformin and the clinically acceptable anti-cancer dosage, we still believe that metformin should be the first choice as a safe adjuvant drug that can indirectly improve the overall survival of cancer patients with existing indications of metformin use. Nonetheless, the available evidence does not yet support metformin as an independent protective factor or prophylactic for melanoma.

## Study limitations

Given some limitations, the proper interpretation of our results requires caution. First, high heterogeneity was observed in the OS, RFS, and incidence analyses, and different study designs, metformin dosage, follow-up duration, drug combinations, previous history, and potential behavioral interventions in the control group may be potential sources of heterogeneity. Due to limitations in the number of included studies and the amount of information provided, we could not conduct an adequate subgroup analysis. Secondly, most of the included studies were non-randomized controlled trials, and these trial data had inherent weaknesses, including potential measurement errors, differences in the definition of drug use, and other risks that could affect the evaluation of the efficacy of metformin. Third, whether enrolled patients were previously treated with tumor-specific therapy (e.g., chemotherapy, radiotherapy, and immune checkpoint inhibitors) or with other non-tumor-specific therapies or interventions may be a confounder in the study of metformin’s efficacy and safety, and increasing inter-study heterogeneity. However, limited by the quantity and quality of current researches in this field, we were unable to draw more accurate conclusions by excluding studies or subgroup analyses. More prospective large cohorts were expected to further investigate the anticancer efficacy of metformin after limiting the patient’s treatment history. Finally, most current studies have focused on the effects of metformin in patients with concurrent diabetes, so it is uncertain whether our findings can be extrapolated to clinically and biologically heterogeneous patients with widespread melanoma.

## Conclusion

Based on the available evidence, we believe that metformin is a safe adjunct that can indirectly improve overall survival in patients with melanoma who meet their clinical indications. However, our findings did not support the hypothesis that metformin is an independent protective factor for melanoma.

## Data availability statement

The original contributions presented in the study are included in the article/**Supplementary Material**. Further inquiries can be directed to the corresponding author.

## Author contributions

HF: Conceptualization, Methodology, Writing – original draft. SS: Conceptualization, Data curation, Writing – review & editing. KC: Conceptualization, Methodology, Writing – review & editing. XS: Conceptualization, Methodology, Writing – review & editing. XY: Conceptualization, Formal Analysis, Writing – review & editing.
