# Supplementary material for: Impact of metformin on melanoma: a meta-analysis and systematic review
Source: Front Oncol. 2024 May 23;14:1399693. doi: 10.3389/fonc.2024.1399693 (PMC11153730; doi:10.3389/fonc.2024.1399693)
Supplement: Supplementary file 1 [file Table_1.docx]

**Search strategy of PubMed**

| NO. | Search Details | Results |
| --- | --- | --- |
| #6 | (#1 OR #3) AND (#2 OR #4) Filters: Humans | 539 |
| #5 | (#1 OR #3) AND (#2 OR #4) | 867 |
| #4 | (((((((((((((((((((((((((((((((((((((((((((((((((((((((((((((((((((((((((((((((((((((((((((((((((((((((((((((((((((((((((((((Dimethylbiguanidine) OR (Dimethylguanylguanidine)) OR (Glucophage)) OR (Metformin Hydrochloride)) OR (Metformin HCl)) OR (anj 900)) OR (anj900)) OR (apophage)) OR (aron)) OR (benofomin)) OR (dabex)) OR (denkaform)) OR (deson)) OR (dextin)) OR (diabetase)) OR (diabetases)) OR (diabetformin)) OR (diabetmin)) OR (diabetmin retard)) OR (diabetosan)) OR (diabex)) OR (diafat)) OR (diaformin)) OR (diaformina)) OR (diaformina lp)) OR (diametin)) OR (diamin)) OR (dianben)) OR (diformin)) OR (diformin retard)) OR (dimefor)) OR (dimethylbiguanide)) OR (dimethyldiguanide)) OR (dmgg)) OR (dybis)) OR (efb 0027)) OR (efb0027)) OR (eraphage)) OR (espa-formin)) OR (euform retard)) OR (fluamine)) OR (flumamine)) OR (fornidd)) OR (fortamet)) OR (glafornil)) OR (glibudon)) OR (glifage)) OR (gliguanid)) OR (glucaminol)) OR (glucofage)) OR (glucofago)) OR (glucoform)) OR (glucoformin)) OR (glucohexal)) OR (glucoless)) OR (glucomet)) OR (glucomin)) OR (glucomine)) OR (gluconil)) OR (glucophage forte)) OR (glucophage retard)) OR (glucophage sr)) OR (glucophage xr)) OR (glucophage xr extended release)) OR (glucophage-mite)) OR (glucostop)) OR (glucotika)) OR (gludepatic)) OR (glufor)) OR (gluformin)) OR (glukophage)) OR (glumeformin)) OR (glumet)) OR (glumetza)) OR (glupa)) OR (glustress)) OR (glyciphage)) OR (glycomet)) OR (glycon)) OR (glycoran)) OR (glyformin)) OR (glymet)) OR (haurymellin)) OR (hipoglucin)) OR (i-max)) OR (islotin)) OR (jesacrin)) OR (juformin)) OR (la 6023)) OR (la6023)) OR (lyomet)) OR (maformin)) OR (meglucon)) OR (meguan)) OR (melbin)) OR (melformin)) OR (mellittin)) OR (merckformin)) OR (mescorit)) OR (metaformin)) OR (metfogamma)) OR (metfoliquid geriasan)) OR (metforal)) OR (metformax)) OR (metformina)) OR (metformine)) OR (metformine hcl)) OR (methformin)) OR (metiguanide)) OR (metomin)) OR (metphormin)) OR (miformin)) OR (neoform)) OR (newmet)) OR (nndg)) OR (reglus-500)) OR (riomet)) OR (riomet er)) OR (risidon)) OR (rudimet)) OR (siamformet)) OR (siofor)) OR (thiabet)) OR (vimetrol)) OR (walaphage)) OR (metformin) | 246,345 |
| #3 | ((((((((((((((Melanomas) OR (Malignant Melanoma)) OR (Malignant Melanomas)) OR (fortner melanoma)) OR (melano-carcinoma)) OR (melano-sarcoma)) OR (melanoblastoma)) OR (melanocarcinoma)) OR (melanocytic malignancies)) OR (melanocytic malignancy)) OR (melanoma)) OR (melanomalignoma)) OR (melanosarcoma)) OR (melanotic carcinoma)) OR (pigmentary cancer) | 167,389 |
| #2 | "Metformin"[Mesh] | 18,140 |
| #1 | "Melanoma"[Mesh] | 110,007 |

**Search strategy of EMBASE**

| No. | Query | Results |
| --- | --- | --- |
| #6 | #5 AND 'human'/de | 576 |
| #5 | (#1 OR #2) AND (#3 OR #4) | 654 |
| #4 | 'dimethylbiguanidine':ti,ab,kw OR 'dimethylguanylguanidine':ti,ab,kw OR 'glucophage':ti,ab,kw OR 'metformin hydrochloride':ti,ab,kw OR 'metformin hcl':ti,ab,kw OR 'anj 900':ti,ab,kw OR 'anj900':ti,ab,kw OR 'apophage':ti,ab,kw OR 'aron':ti,ab,kw OR 'benofomin':ti,ab,kw OR 'dabex':ti,ab,kw OR 'denkaform':ti,ab,kw OR 'deson':ti,ab,kw OR 'dextin':ti,ab,kw OR 'diabetase':ti,ab,kw OR 'diabetases':ti,ab,kw OR 'diabetformin':ti,ab,kw OR 'diabetmin':ti,ab,kw OR 'diabetmin retard':ti,ab,kw OR 'diabetosan':ti,ab,kw OR 'diabex':ti,ab,kw OR 'diafat':ti,ab,kw OR 'diaformin':ti,ab,kw OR 'diaformina':ti,ab,kw OR 'diaformina lp':ti,ab,kw OR 'diametin':ti,ab,kw OR 'diamin':ti,ab,kw OR 'dianben':ti,ab,kw OR 'diformin':ti,ab,kw OR 'diformin retard':ti,ab,kw OR 'dimefor':ti,ab,kw OR 'dimethylbiguanide':ti,ab,kw OR 'dimethyldiguanide':ti,ab,kw OR 'dmgg':ti,ab,kw OR 'dybis':ti,ab,kw OR 'efb 0027':ti,ab,kw OR 'efb0027':ti,ab,kw OR 'eraphage':ti,ab,kw OR 'espa-formin':ti,ab,kw OR 'euform retard':ti,ab,kw OR 'fluamine':ti,ab,kw OR 'flumamine':ti,ab,kw OR 'fornidd':ti,ab,kw OR 'fortamet':ti,ab,kw OR 'glafornil':ti,ab,kw OR 'glibudon':ti,ab,kw OR 'glifage':ti,ab,kw OR 'gliguanid':ti,ab,kw OR 'glucaminol':ti,ab,kw OR 'glucofage':ti,ab,kw OR 'glucofago':ti,ab,kw OR 'glucoform':ti,ab,kw OR 'glucoformin':ti,ab,kw OR 'glucohexal':ti,ab,kw OR 'glucoless':ti,ab,kw OR 'glucomet':ti,ab,kw OR 'glucomin':ti,ab,kw OR 'glucomine':ti,ab,kw OR 'gluconil':ti,ab,kw OR 'glucophage forte':ti,ab,kw OR 'glucophage retard':ti,ab,kw OR 'glucophage sr':ti,ab,kw OR 'glucophage xr':ti,ab,kw OR 'glucophage xr extended release':ti,ab,kw OR 'glucophage-mite':ti,ab,kw OR 'glucostop':ti,ab,kw OR 'glucotika':ti,ab,kw OR 'gludepatic':ti,ab,kw OR 'glufor':ti,ab,kw OR 'gluformin':ti,ab,kw OR 'glukophage':ti,ab,kw OR 'glumeformin':ti,ab,kw OR 'glumet':ti,ab,kw OR 'glumetza':ti,ab,kw OR 'glupa':ti,ab,kw OR 'glustress':ti,ab,kw OR 'glyciphage':ti,ab,kw OR 'glycomet':ti,ab,kw OR 'glycon':ti,ab,kw OR 'glycoran':ti,ab,kw OR 'glyformin':ti,ab,kw OR 'glymet':ti,ab,kw OR 'haurymellin':ti,ab,kw OR 'hipoglucin':ti,ab,kw OR 'i-max':ti,ab,kw OR 'islotin':ti,ab,kw OR 'jesacrin':ti,ab,kw OR 'juformin':ti,ab,kw OR 'la 6023':ti,ab,kw OR 'la6023':ti,ab,kw OR 'lyomet':ti,ab,kw OR 'maformin':ti,ab,kw OR 'meglucon':ti,ab,kw OR 'meguan':ti,ab,kw OR 'melbin':ti,ab,kw OR 'melformin':ti,ab,kw OR 'mellittin':ti,ab,kw OR 'merckformin':ti,ab,kw OR 'mescorit':ti,ab,kw OR 'metaformin':ti,ab,kw OR 'metfogamma':ti,ab,kw OR 'metfoliquid geriasan':ti,ab,kw OR 'metforal':ti,ab,kw OR 'metformax':ti,ab,kw OR 'metformina':ti,ab,kw OR 'metformine':ti,ab,kw OR 'metformine hcl':ti,ab,kw OR 'methformin':ti,ab,kw OR 'metiguanide':ti,ab,kw OR 'metomin':ti,ab,kw OR 'metphormin':ti,ab,kw OR 'miformin':ti,ab,kw OR 'neoform':ti,ab,kw OR 'newmet':ti,ab,kw OR 'nndg':ti,ab,kw OR 'reglus-500':ti,ab,kw OR 'riomet':ti,ab,kw OR 'riomet er':ti,ab,kw OR 'risidon':ti,ab,kw OR 'rudimet':ti,ab,kw OR 'siamformet':ti,ab,kw OR 'siofor':ti,ab,kw OR 'thiabet':ti,ab,kw OR 'vimetrol':ti,ab,kw OR 'walaphage':ti,ab,kw OR 'metformin':ti,ab,kw | 50482 |
| #3 | 'metformin'/exp | 88474 |
| #2 | 'melanomas':ti,ab,kw OR 'malignant melanoma':ti,ab,kw OR 'malignant melanomas':ti,ab,kw OR 'fortner melanoma':ti,ab,kw OR 'melano-carcinoma':ti,ab,kw OR 'melano-sarcoma':ti,ab,kw OR 'melanoblastoma':ti,ab,kw OR 'melanocarcinoma':ti,ab,kw OR 'melanocytic malignancies':ti,ab,kw OR 'melanocytic malignancy':ti,ab,kw OR 'melanoma':ti,ab,kw OR 'melanomalignoma':ti,ab,kw OR 'melanosarcoma':ti,ab,kw OR 'melanotic carcinoma':ti,ab,kw OR 'pigmentary cancer':ti,ab,kw | 205788 |
| #1 | 'melanoma'/exp | 210455 |

**Search strategy of Cochrane Library**

| NO. | Search deatiles | Hits |
| --- | --- | --- |
| #1 | MeSH descriptor: [Melanoma] explode all trees | 2761 |
| #2 | (Melanomas):ti,ab,kw OR (Malignant Melanoma):ti,ab,kw OR (Malignant Melanomas):ti,ab,kw OR (fortner melanoma):ti,ab,kw OR (melano-carcinoma):ti,ab,kw OR (melano-sarcoma):ti,ab,kw OR (melanoblastoma):ti,ab,kw OR (melanocarcinoma):ti,ab,kw OR (melanocytic malignancies):ti,ab,kw OR (melanocytic malignancy):ti,ab,kw OR (melanoma):ti,ab,kw OR (melanomalignoma):ti,ab,kw OR (melanosarcoma):ti,ab,kw OR (melanotic carcinoma):ti,ab,kw OR (pigmentary cancer):ti,ab,kw | 6566 |
| #3 | MeSH descriptor: [Metformin] explode all trees | 4991 |
| #4 | (Dimethylbiguanidine):ti,ab,kw OR (Dimethylguanylguanidine):ti,ab,kw OR (Glucophage):ti,ab,kw OR (Metformin Hydrochloride):ti,ab,kw OR (Metformin HCl):ti,ab,kw OR (anj 900):ti,ab,kw OR (anj900):ti,ab,kw OR (apophage):ti,ab,kw OR (aron):ti,ab,kw OR (benofomin):ti,ab,kw OR (dabex):ti,ab,kw OR (denkaform):ti,ab,kw OR (deson):ti,ab,kw OR (dextin):ti,ab,kw OR (diabetase):ti,ab,kw OR (diabetases):ti,ab,kw OR (diabetformin):ti,ab,kw OR (diabetmin):ti,ab,kw OR (diabetmin retard):ti,ab,kw OR (diabetosan):ti,ab,kw OR (diabex):ti,ab,kw OR (diafat):ti,ab,kw OR (diaformin):ti,ab,kw OR (diaformina):ti,ab,kw OR (diaformina lp):ti,ab,kw OR (diametin):ti,ab,kw OR (diamin):ti,ab,kw OR (dianben):ti,ab,kw OR (diformin):ti,ab,kw OR (diformin retard):ti,ab,kw OR (dimefor):ti,ab,kw OR (dimethylbiguanide):ti,ab,kw OR (dimethyldiguanide):ti,ab,kw OR (dmgg):ti,ab,kw OR (dybis):ti,ab,kw OR (efb 0027):ti,ab,kw OR (efb0027):ti,ab,kw OR (eraphage):ti,ab,kw OR (espa-formin):ti,ab,kw OR (euform retard):ti,ab,kw OR (fluamine):ti,ab,kw OR (flumamine):ti,ab,kw OR (fornidd):ti,ab,kw OR (fortamet):ti,ab,kw OR (glafornil):ti,ab,kw OR (glibudon):ti,ab,kw OR (glifage):ti,ab,kw OR (gliguanid):ti,ab,kw OR (glucaminol):ti,ab,kw OR (glucofage):ti,ab,kw OR (glucofago):ti,ab,kw OR (glucoform):ti,ab,kw OR (glucoformin):ti,ab,kw OR (glucohexal):ti,ab,kw OR (glucoless):ti,ab,kw OR (glucomet):ti,ab,kw OR (glucomin):ti,ab,kw OR (glucomine):ti,ab,kw OR (gluconil):ti,ab,kw OR (glucophage forte):ti,ab,kw OR (glucophage retard):ti,ab,kw OR (glucophage sr):ti,ab,kw OR (glucophage xr):ti,ab,kw OR (glucophage xr extended release):ti,ab,kw OR (glucophage-mite):ti,ab,kw OR (glucostop):ti,ab,kw OR (glucotika):ti,ab,kw OR (gludepatic):ti,ab,kw OR (glufor):ti,ab,kw OR (gluformin):ti,ab,kw OR (glukophage):ti,ab,kw OR (glumeformin):ti,ab,kw OR (glumet):ti,ab,kw OR (glumetza):ti,ab,kw OR (glupa):ti,ab,kw OR (glustress):ti,ab,kw OR (glyciphage):ti,ab,kw OR (glycomet):ti,ab,kw OR (glycon):ti,ab,kw OR (glycoran):ti,ab,kw OR (glyformin):ti,ab,kw OR (glymet):ti,ab,kw OR (haurymellin):ti,ab,kw OR (hipoglucin):ti,ab,kw OR (i-max):ti,ab,kw OR (islotin):ti,ab,kw OR (jesacrin):ti,ab,kw OR (juformin):ti,ab,kw OR (la 6023):ti,ab,kw OR (la6023):ti,ab,kw OR (lyomet):ti,ab,kw OR (maformin):ti,ab,kw OR (meglucon):ti,ab,kw OR (meguan):ti,ab,kw OR (melbin):ti,ab,kw OR (melformin):ti,ab,kw OR (mellittin):ti,ab,kw OR (merckformin):ti,ab,kw OR (mescorit):ti,ab,kw OR (metaformin):ti,ab,kw OR (metfogamma):ti,ab,kw OR (metfoliquid geriasan):ti,ab,kw OR (metforal):ti,ab,kw OR (metformax):ti,ab,kw OR (metformina):ti,ab,kw OR (metformine):ti,ab,kw OR (metformine hcl):ti,ab,kw OR (methformin):ti,ab,kw OR (metiguanide):ti,ab,kw OR (metomin):ti,ab,kw OR (metphormin):ti,ab,kw OR (miformin):ti,ab,kw OR (neoform):ti,ab,kw OR (newmet):ti,ab,kw OR (nndg):ti,ab,kw OR (reglus-500):ti,ab,kw OR (riomet):ti,ab,kw OR (riomet er):ti,ab,kw OR (risidon):ti,ab,kw OR (rudimet):ti,ab,kw OR (siamformet):ti,ab,kw OR (siofor):ti,ab,kw OR (thiabet):ti,ab,kw OR (vimetrol):ti,ab,kw OR (walaphage):ti,ab,kw OR (metformin):ti,ab,kw | 13152 |
| #5 | (#1 OR #2) AND (#3 OR #4) | 14 |

**Search strategy of Web of science**

| NO. | Search deatiles | Hits |
| --- | --- | --- |
| #1 | (((((((((((((TS=(Melanomas) OR TS=(Malignant Melanoma)) OR TS=(Malignant Melanomas)) OR TS=(fortner melanoma)) OR TS=(melano-carcinoma)) OR TS=(melano-sarcoma)) OR TS=(melanoblastoma)) OR TS=(melanocarcinoma)) OR TS=(melanocytic malignancies)) OR TS=(melanocytic malignancy)) OR TS=(melanoma)) OR TS=(melanomalignoma)) OR TS=(melanosarcoma)) OR TS=(melanotic carcinoma)) OR TS=(pigmentary cancer) | 195106 |
| #2 | ((((((((((((((((((((((((((((((((((((((((((((((((((((((((((((((((((((((((((((((((((((((((((((((((((((((((((((((((((((((((((((TS=(Dimethylbiguanidine) OR TS=(Dimethylguanylguanidine)) OR TS=(Glucophage)) OR TS=(Metformin Hydrochloride)) OR TS=(Metformin HCl)) OR TS=(anj 900)) OR TS=(anj900)) OR TS=(apophage)) OR TS=(aron)) OR TS=(benofomin)) OR TS=(dabex)) OR TS=(denkaform)) OR TS=(deson)) OR TS=(dextin)) OR TS=(diabetase)) OR TS=(diabetases)) OR TS=(diabetformin)) OR TS=(diabetmin)) OR TS=(diabetmin retard)) OR TS=(diabetosan)) OR TS=(diabex)) OR TS=(diafat)) OR TS=(diaformin)) OR TS=(diaformina)) OR TS=(diaformina lp)) OR TS=(diametin)) OR TS=(diamin)) OR TS=(dianben)) OR TS=(diformin)) OR TS=(diformin retard)) OR TS=(dimefor)) OR TS=(dimethylbiguanide)) OR TS=(dimethyldiguanide)) OR TS=(dmgg)) OR TS=(dybis)) OR TS=(efb 0027)) OR TS=(efb0027)) OR TS=(eraphage)) OR TS=(espa-formin)) OR TS=(euform retard)) OR TS=(fluamine)) OR TS=(flumamine)) OR TS=(fornidd)) OR TS=(fortamet)) OR TS=(glafornil)) OR TS=(glibudon)) OR TS=(glifage)) OR TS=(gliguanid)) OR TS=(glucaminol)) OR TS=(glucofage)) OR TS=(glucofago)) OR TS=(glucoform)) OR TS=(glucoformin)) OR TS=(glucohexal)) OR TS=(glucoless)) OR TS=(glucomet)) OR TS=(glucomin)) OR TS=(glucomine)) OR TS=(gluconil)) OR TS=(glucophage forte)) OR TS=(glucophage retard)) OR TS=(glucophage sr)) OR TS=(glucophage xr)) OR TS=(glucophage xr extended release)) OR TS=(glucophage-mite)) OR TS=(glucostop)) OR TS=(glucotika)) OR TS=(gludepatic)) OR TS=(glufor)) OR TS=(gluformin)) OR TS=(glukophage)) OR TS=(glumeformin)) OR TS=(glumet)) OR TS=(glumetza)) OR TS=(glupa)) OR TS=(glustress)) OR TS=(glyciphage)) OR TS=(glycomet)) OR TS=(glycon)) OR TS=(glycoran)) OR TS=(glyformin)) OR TS=(glymet)) OR TS=(haurymellin)) OR TS=(hipoglucin)) OR TS=(i-max)) OR TS=(islotin)) OR TS=(jesacrin)) OR TS=(juformin)) OR TS=(la 6023)) OR TS=(la6023)) OR TS=(lyomet)) OR TS=(maformin)) OR TS=(meglucon)) OR TS=(meguan)) OR TS=(melbin)) OR TS=(melformin)) OR TS=(mellittin)) OR TS=(merckformin)) OR TS=(mescorit)) OR TS=(metaformin)) OR TS=(metfogamma)) OR TS=(metfoliquid geriasan)) OR TS=(metforal)) OR TS=(metformax)) OR TS=(metformina)) OR TS=(metformine)) OR TS=(metformine hcl)) OR TS=(methformin)) OR TS=(metiguanide)) OR TS=(metomin)) OR TS=(metphormin)) OR TS=(miformin)) OR TS=(neoform)) OR TS=(newmet)) OR TS=(nndg)) OR TS=(reglus-500)) OR TS=(riomet)) OR TS=(riomet er)) OR TS=(risidon)) OR TS=(rudimet)) OR TS=(siamformet)) OR TS=(siofor)) OR TS=(thiabet)) OR TS=(vimetrol)) OR TS=(walaphage)) OR TS=(metformin) | 44358 |
| #3 | #2 AND #1 | 172 |
